# Flash Pulmonary Edema From Brief Loss of Biventricular Pacing During CRT-D Generator Exchange

**DOI:** 10.1016/j.jaccas.2026.107942

**Published:** 2026-04-16

**Authors:** Joud Fahed, Resha Reya Ganthan, Rana Al-Zakhari, Rayna Isber, Nidal Isber

**Affiliations:** a Department of Internal Medicine, Ascension Saint Agnes Hospital, Baltimore, Maryland, USA; b Department of Internal Medicine, Richmond University Medical Center/Mount Sinai, Staten Island, New York, USA; c Department of Biology, Barnard College, Manhattan, New York, USA; d Department of Electrophysiology, Richmond University Medical Center/Mount Sinai, Staten Island, New York, USA

**Keywords:** acute pulmonary edema, biventricular pacing, cardiac resynchronization therapy, flash pulmonary edema, generator change

## Abstract

**Background:**

Acute cardiogenic pulmonary edema results from abrupt cardiopulmonary derangements, including arrhythmia or acute valvular dysfunction.

**Case Summary:**

An 88-year-old man with nonischemic cardiomyopathy, NYHA functional class III heart failure, left bundle branch block, and severe mitral regurgitation underwent biventricular implantable cardioverter-defibrillator generator replacement. During the procedure, biventricular pacing was interrupted. Within minutes, he developed acute dyspnea, agitation, and hypoxia consistent with flash pulmonary edema.

**Discussion:**

Loss of biventricular pacing likely caused ventricular dyssynchrony, worsening mitral regurgitation, and rapidly increasing pulmonary venous pressure. Restoration of pacing improved symptoms.

**Take-Home Messages:**

Interruption of biventricular pacing during generator exchange can precipitate flash pulmonary edema, especially in patients with significant mitral regurgitation. Ventricular dyssynchrony from pacing cessation may acutely worsen mitral regurgitation, leading to rapid pulmonary congestion.

## Graphical Abstract

**Figure:**
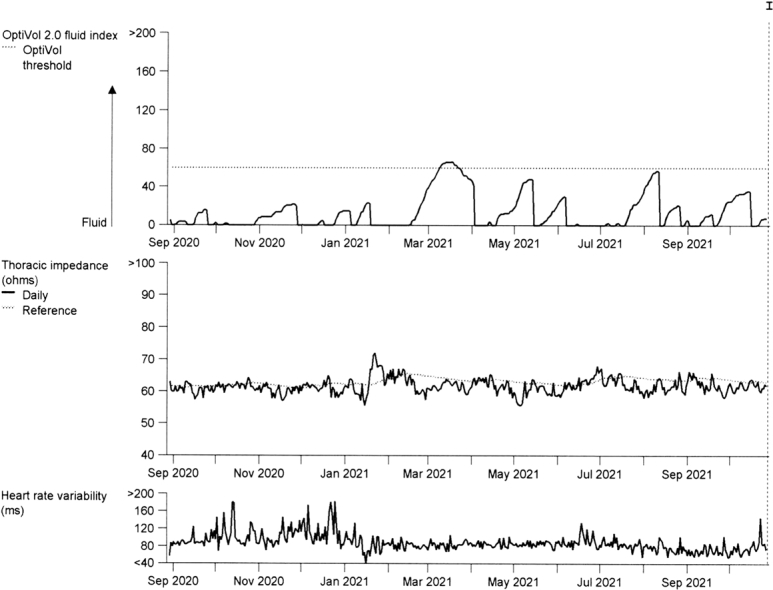

Sudden onset of cardiogenic pulmonary edema can be referred to as flash pulmonary edema (FPE), which is caused by an abrupt physiologic instability such as acute onset of hypertension, acute myocarditis, arrhythmia, acute myocardial ischemia, or acute valvular dysfunction such as in mitral regurgitation (MR).^1^ FPE results from rapid elevation of left-sided cardiac filling pressures, leading to increased pulmonary capillary hydrostatic pressure and fluid transudation into the alveolar interstitium.^2^

**Take-Home Messages**

• Cessation of biventricular pacing during biventricular implantable cardioverter-defibrillator generator change can lead to flash pulmonary edema, especially in patients with pre-existing mitral regurgitation.
• Sudden discontinuation of biventricular pacing can predispose to ventricular dyssynchrony, which is thought to exacerbate regurgitation of the mitral valve. This exacerbation results in rapid increase in pulmonary venous pressure leading to acute pulmonary edema.

Sudden ventricular dyssynchrony secondary to left bundle branch block (LBBB) is not a well-known cause of FPE. However, LBBB is known to impair left ventricular (LV) systolic performance and exacerbate functional MR through discoordinated papillary muscle activation and reduction in LV closing force.^3^^,^^4^ Cardiac resynchronization therapy (CRT) improves ventricular synchrony, enhances contractile efficiency, and has been shown to reduce functional MR severity in appropriately selected heart failure patients.^5^, ^6^, ^7^, ^8^, ^9^

Herein, we present a case in which FPE occurred during a biventricular defibrillator generator change, minutes following the disconnection of biventricular implantable cardioverter-defibrillator (BiV-ICD) leads, which resulted in sudden resumption of conduction with LBBB and ventricular dyssynchrony that worsened the degree of MR, resulting in FPE.

## History of Presentation

An 88-year-old man who was previously diagnosed with hypertension, nonischemic cardiomyopathy, NYHA class III congestive heart failure, LBBB, and grade 3 MR received a BiV-ICD device in addition to optimal medical therapy. His medications included carvedilol, furosemide, spironolactone, sacubitril/valsartan, and dapagliflozin. The patient was managed successfully for years without need of hospitalization for heart failure exacerbation.

The patient presented to our electrophysiology laboratory for BiV-ICD battery change after his device had reached the elective replacement time. Preadmission assessment and testing showed that he was clinically stable; he had no symptoms including shortness of breath, cough, chest pain, dizziness, or palpitations. He could manage his daily activities without difficulty, and he could walk 2 to 3 city blocks and climb a flight of stairs without becoming dyspneic.

**Figure 1:**
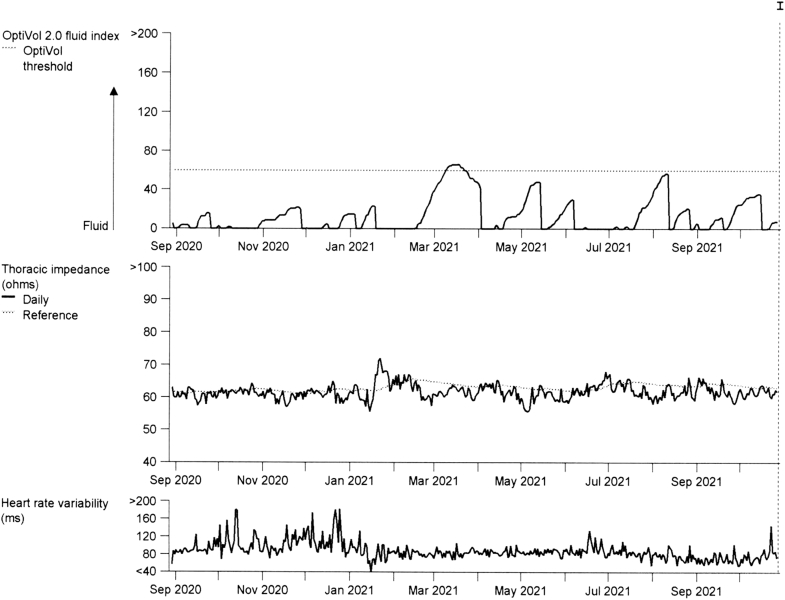
Preprocedural Biventricular ICD Device Interrogation Demonstrating Stable Transthoracic Impedance and OptiVol 2.0 Fluid Index Values, With No Evidence of Elevated Intrathoracic Fluid or Pulmonary Congestion ICD = implantable cardioverter-defibrillator.

His electrocardiogram showed sinus rhythm with biventricular pacing. The BiV-ICD device interrogation confirmed that the battery was reaching the elective replacement time, and the transthoracic impedance and OptiVol fluid index revealed no current pulmonary congestion (Figure 1).

Under local anesthesia only, the BiV-ICD pocket was opened, and the device was extracted from the pocket and disconnected from the leads. Several minutes after disconnection while the pocket was being remodeled and hemostasis was being obtained, the patient started to become dyspneic, anxious, and confused. His oxygen saturation began to drop, and he began moving. It was immediately realized that cessation of biventricular pacing might have contributed to the patient's sudden decompensation; therefore, a new BiV-ICD generator was promptly connected to the leads to restore ventricular resynchronization.

The patient's oxygen saturation continued to drop while he became cyanotic, progressively agitated, and finally somnolent. Upon visual inspection, he was dyspneic, drowsy, pale, diaphoretic, and restless. Blood pressure was measured at 142/82 mm Hg, and rhythm was sinus tachycardia at 120 beats/min. No sustained ventricular arrhythmias were observed. There was no documented hypertensive crisis, no chest pain suggestive of acute ischemia, and no significant periprocedural intravenous fluid administration beyond minimal flushes. The initial oxygen saturation was 75% on high-flow oxygen via nasal cannula. Respiratory rate was 35 breaths/min with diffuse bilateral rhonchi, rales, and wheezing was audible from several feet away.

The patient was placed in the seated position. His nasal cannula was replaced by nonrebreather mask and subsequently by bilevel positive airway pressure. Arterial blood gas analysis was not obtained during the acute episode because of the rapidly evolving respiratory distress and the immediate initiation of noninvasive ventilatory support. Clinical management priorities focused on prompt stabilization of oxygenation and respiratory mechanics, and the patient demonstrated rapid clinical improvement after bilevel positive airway pressure therapy, which limited the opportunity for additional invasive diagnostic testing.

**Figure 2:**
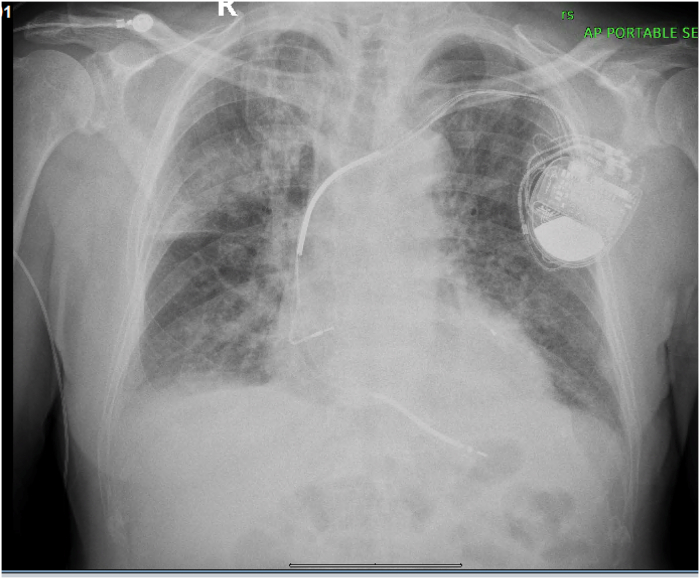
Portable Anteroposterior Chest Radiograph Showing Bilateral Alveolar-Interstitial Infiltrates Predominantly in the Perihilar and Lower Lung Zones, Consistent With Acute Pulmonary Edema

Medical therapy was initiated, including intravenous nitroglycerin infusion titrated to blood pressure and repeated doses of intravenous furosemide guided by clinical response and urine output. Chest x-ray revealed bilateral alveolar-interstitial infiltrates consistent with pulmonary congestion (Figure 2). Medical therapy resulted in rapid clinical improvement, allowing completion of the procedure.

## Outcome and Follow-Up

One week after discharge, the patient was seen in the cardiology clinic and found to be stable with NYHA functional class II heart failure. Blood pressure was 120/74 mm Hg and pulse rate was 70 beats/min. Medications were uptitrated.

**Figure 3:**
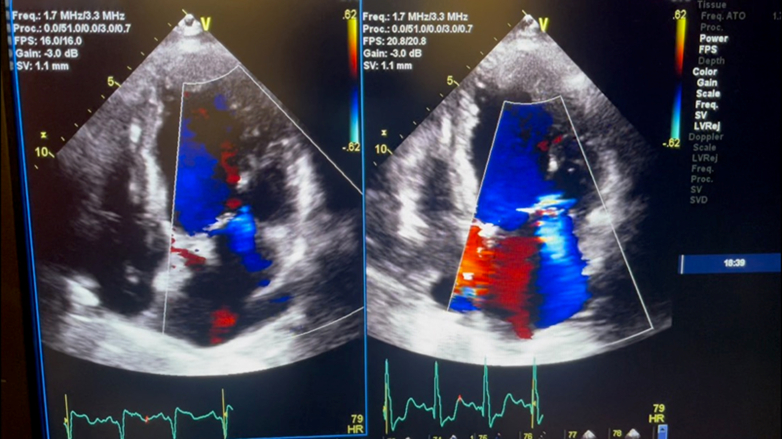
Transthoracic Echocardiogram in the Apical 4-Chamber View Illustrating Severe Left Ventricular Systolic Dysfunction With an Ejection Fraction of Approximately 25% Under biventricular pacing (Left), only mild (grade 1) mitral regurgitation was observed. However, temporary discontinuation of pacing (Right) led to ventricular dyssynchrony and a rapid increase in regurgitant jet severity to grade 2-3.

To investigate the etiology of FPE and to evaluate the hypothesis that temporary discontinuation of biventricular pacing may have contributed to ventricular dysfunction, transthoracic echocardiography was performed. MR was grade 1 during biventricular pacing; however, when biventricular pacing was temporarily deactivated, MR worsened to grade 2-3 (Figure 3).

Although quantitative parameters such as effective regurgitant orifice area, regurgitant volume, vena contracta width, pulmonary vein Doppler patterns, or LV contractility (dP/dt) were not formally measured, semiquantitative assessment demonstrated increased regurgitant jet area and systolic leaflet malcoaptation when resynchronization was interrupted.

This comparison was performed after clinical stabilization and is consistent with the hypothesis that loss of CRT may dynamically worsen functional MR in susceptible patients.

## Discussion

Acute cardiogenic pulmonary edema, or FPE, is a potentially fatal condition caused by excessive accumulation of fluid in the alveolar spaces owing to disturbance in pulmonary Starling forces.^2^ The proposed pathophysiologic mechanism in the present case is inferential because invasive hemodynamic monitoring and real-time echocardiographic imaging were not obtained during the acute episode. Although causality cannot be definitively established, the temporal relationship between pacing interruption and respiratory deterioration, absence of alternative clear precipitants, and reproducible worsening of MR when pacing was temporarily deactivated provide physiologic plausibility.

Evaluation and management of FPE should focus on stabilization of respiratory distress followed by identification of underlying etiology. Causes of cardiogenic pulmonary edema include congestive heart failure, cardiomyopathies, left-sided valvular disease, coronary artery disease with ventricular failure, shunt physiology, and cardiac arrhythmias.^2^

As in the present case, FPE may be associated with LBBB in patients with LV systolic dysfunction. Ventricular dyssynchrony secondary to LBBB can contribute to development or worsening of functional MR.^3^^,^^4^

Under normal electrical activation, papillary muscles are activated prior to LV free wall contraction, allowing chordal tensioning that maintains mitral leaflet coaptation. Dyssynchronous activation disrupts coordinated papillary muscle contraction, reducing mitral valve competence. In heart failure, increased leaflet tethering forces impair valve closure, and mitral competence becomes dependent on early systolic LV–left atrial pressure gradients, which are reduced in LBBB given delayed LV pressure rise.^3^ LBBB may also promote adverse ventricular remodeling with apical and lateral papillary muscle displacement, further impairing mitral valve function.^3^^,^^4^

Cardiac resynchronization therapy is a Class I recommendation for symptomatic heart failure patients with LV ejection fraction ≤35%, QRS duration ≥150 ms, and LBBB morphology despite optimal medical therapy.^5^ CRT has been associated with reduction of significant MR burden in approximately 34% of patients after sustained therapy.^6^

**Figure 4:**
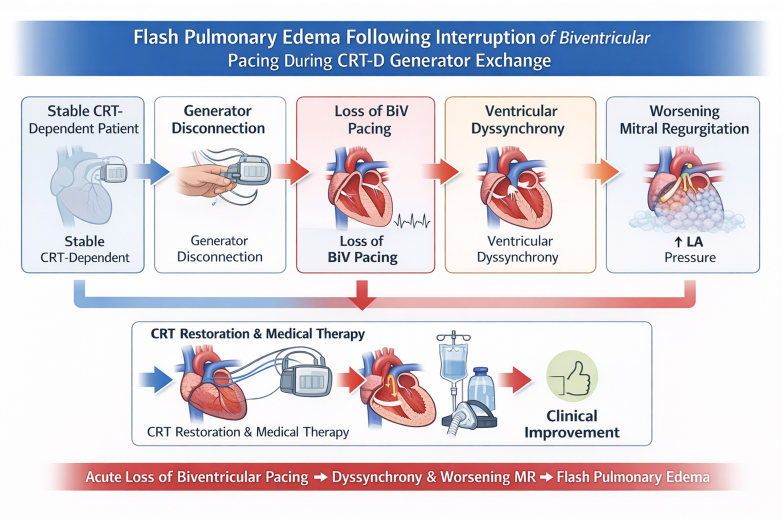
Central Illustration: Flash Pulmonary Edema Following Interruption of Biventricular Pacing During CRT-D Generator Exchange Temporary interruption of biventricular pacing during CRT-D generator exchange resulted in reversion to intrinsic conduction with left bundle branch block and acute ventricular dyssynchrony. Dyssynchrony likely exacerbated functional mitral regurgitation, increasing left atrial pressure and precipitating flash pulmonary edema. Restoration of CRT was associated with rapid hemodynamic and clinical improvement. BiV = biventricular; CRT-D = cardiac resynchronization therapy device; LA = left atrial; MR = mitral regurgitation.

The degree of MR in this patient is presumed to have been minimized by long-term CRT therapy, and interruption of pacing may have resulted in acute worsening of pre-existing functional MR. The mechanism of MR reduction after CRT is believed to relate to improvement in LV closing force and transmitral pressure gradient generation.^7^ The proposed mechanism is summarized in the Central Illustration (Figure 4).

Mechanical activation strain mapping studies demonstrate that CRT can produce immediate MR reduction by improving synchrony of papillary muscle insertion site activation.^8^ In the CARE-HF trial of 813 patients, CRT therapy was associated with greater MR reduction compared with optimized medical therapy alone.^9^ Patients with MR reduction after CRT have demonstrated improved survival during follow-up.^10^^,^^11^

### Alternative mechanisms considered

Acute pulmonary edema during device procedures may occur because of transient hypertensive response, sympathetic activation, myocardial ischemia, arrhythmic instability, or dynamic preload/afterload changes.^1^, ^2^, ^3^

In this patient, blood pressure elevation was modest, no ischemic symptoms were reported, no sustained arrhythmias were documented, and fluid administration was minimal. Although sympathetic activation related to procedural stress cannot be excluded, the abrupt onset after pacing interruption and rapid improvement after CRT restoration support dyssynchrony-related MR worsening as a likely contributor. Multifactorial pathophysiology remains possible.

This case contributes clinically by highlighting a potential procedural vulnerability during CRT device generator exchange, emphasizing that even brief interruption of chronic biventricular pacing may have hemodynamic consequences in selected high-risk patients with severe functional MR and advanced systolic dysfunction.

### Limitations

This report is limited by the absence of invasive hemodynamic measurements, lack of real-time imaging during acute deterioration, and absence of quantitative MR metrics. Semiquantitative echocardiographic assessment was operator dependent. Therefore, the proposed mechanism should be interpreted as hypothesis-generating rather than definitive.

## Conclusions

Cessation of biventricular pacing during generator exchange of a CRT device may lead to flash pulmonary edema, particularly in patients with pre-existing moderate to severe MR and severe LV dysfunction. While direct causality cannot be conclusively established, selected patients with CRT-responsive functional MR may be vulnerable to even brief interruption of ventricular resynchronization therapy. Efforts should be made to minimize interruption time during generator replacement procedures.

## Funding Support and Author Disclosures

The authors have reported that they have no relationships relevant to the contents of this paper to disclose.
